# The Feasibility of Using *Coxiella burnetii* Avirulent Nine Mile Phase II Viable Bacteria as a Live Attenuated Vaccine Against Q fever

**DOI:** 10.3389/fimmu.2021.754690

**Published:** 2021-10-21

**Authors:** Venkatesh Kumaresan, Shawkat Alam, Yan Zhang, Guoquan Zhang

**Affiliations:** Department of Molecular Microbiology and Immunology, University of Texas at San Antonio, San Antonio, TX, United States

**Keywords:** *Coxiella burnetii*, Nine Mile-Phase I/Phase II LPS, viable avirulent bacteria, live attenuated vaccine, BALB/c mice, humoral and cellular immunity, cross-protection

## Abstract

This study aimed to explore if viable *C. burnetii* avirulent Nine Mile phase II (NMII) can elicit protective immunity against virulent NM phase I (NMI) infection. Interestingly, mice immunized with viable NMII elicited significant protection against NMI infection at different time points post-immunization. Viable NMII induced a dose-dependent NMI-specific IgG response in mice, but all doses of NMII-immunized mice conferred a similar level of protection. Comparing different routes of immunization indicated that intranasally immunized mice showed significantly higher levels of protection than other immunization routes. The observation that viable NMII induced a similar level of long-term protection against NMI challenge as the formalin-inactivated NMI vaccine (PIV) suggests that viable NMII bacteria can induce a similar level of long-term protection against virulent NMI challenge as the PIV. Viable NMII also induced significant protection against challenge with virulent Priscilla and Scurry strains, suggesting that viable NMII can elicit broad protection. Immune sera and splenocytes from viable NMII-immunized mice are protective against NMI infection, but immune serum-receiving mice did not control NMI replication. Additionally, viable NMII conferred a comparable level of protection in wild-type, CD4^+^ T cell-deficient, and CD8^+^ T cell-deficient mice, and partial protection in B cell-deficient mice. However, NMII-immunized T cell-deficient mice were unable to prevent *C. burnetii* replication. Thus, both B cells and T cells are required for viable NMII-induced protective immunity but T cells may play a critical role. Collectively, this study demonstrates the feasibility of using avirulent NMII as a live attenuated vaccine against human Q fever.

## Introduction

*Coxiella burnetii* is an obligate intracellular bacterium that causes flu-like zoonosis and Q-fever. Cattle, sheep, and goat are the primary reservoirs for human infections. The enhanced stability of the organism in aerosol contributes to their potential to initiate a pandemic and therefore classified as a Tier 2 select agent by the United States Centers for Disease Control and Prevention (CDC) (1). Previous reports suggest that intertropical areas are commonly prone to *C. burnetii* infection (2, 3). The Q fever outbreak in Netherlands from 2007 to 2010 resulted in 4,107 notifications, indicating that Q fever remains a significant threat for public health (4, 5). Mostly, serological assays are used to diagnose acute infection and most of these patients recover after treatment with 100 mg dose of doxycycline twice a day for 2 weeks (6). In case of chronic Q-fever, a 100-mg dose of doxycycline was administered twice daily, 200 mg hydroxychloroquine three times a day for a minimum of 18 months and longer in immune-compromised patients (7). To prevent these complications, a wide-range preventive vaccine is critical, specifically for those at risk due to occupation, such as veterinarians, meat-processing plant workers, sheep and dairy workers, livestock farmers, and researchers at facilities housing sheep.

Q-Vax^®^ is the only licensed vaccine for human vaccination, which is a formalin-inactivated whole-cell vaccine, produced from the Henzerling Phase I strain and which can provide reliable protection against Q fever (8). However, the vaccine induces adverse reactions such as subcutis swelling, erythema, and induration in subjects pre-immune to *C. burnetii* (9). Therefore, it is required to test for sensitization to Q fever antigens using Q-VAX^®^ Skin Test in every individual prior to immunization. In terms of veterinary vaccines, Chlamyvax FQ^®^ and Coxevac^®^ are commercially available. Chlamyvax FQ is prepared as an oil emulsion with *Chlamydophila abortus* and inactivated phase II *C. burnetii* and is approved in France (10). However, Chlamyvax FQ did not show effectiveness in protection against abortion and *C. burnetii* shedding in milk, feces, placenta, and vaginal secretions compared to the unvaccinated control group (11). In our lab, we have developed a Phase I LPS-targeted peptide mimic vaccine based on the concept of reverse vaccinology. Although vaccination with the mimotope vaccine elicited significant protection against *C. burnetii* infection in mice, the levels of protection are comparatively lower compared to the formalin-inactivated NMI whole-cell vaccine (PIV) (12), suggesting that other antigenic components beyond LPS in PIV might be necessary for coffering complete protection.

Among the limited virulence factors reported in *C. burnetii*, LPS is the major virulence factor where the O-antigen plays a major role in pathogenesis (13). Moreover, phase I LPS was able to confer protective immunity against virulent *C. burnetii* infection (14). Virulent *C. burnetii* NMI strains undergo phase variation after serial passage in embryonated eggs, which is a non-reversible shift from full-length LPS of virulent NMI bacteria to avirulent NMII with truncated LPS lacking O-antigen and several core sugars (15). Whole-genome sequencing revealed that NMII has a ~26-kb chromosomal deletion, compared to NMI, where the deleted region eliminates several LPS biosynthetic genes resulting in the production of truncated LPS (16–20); therefore, NMII bacteria cannot revert to phase I LPS-expressing *C. burnetii*. Because of clonality, avirulence in a guinea pig model of infection, and lack of phase reversion, the NMII strain is considered as a biosafety level-2 (BSL-2) pathogen (16, 21). Vishwanath and Hackstadt (22) demonstrated that NMI with smooth LPS was resistant to complement-mediated killing by human serum, whereas NMII with rough LPS was killed by serum complement. Our earlier studies indicated that the rate of replication of NMII in mouse immune cells including neutrophils (23) and B cells (24) is different with that of NMI. Infection in BALB/c mice indicated that 10^7^ of NMI bacteria can induce high splenomegaly while 10^7^ of NMII bacteria do not induce splenomegaly or fever in guinea pigs (25). Moos and Hackstadt (26) reported that NMII did not elicit fever or seroconversion except with very large inocula (10^8^), and viable organisms could not be recovered at 30 days postinfection in guinea pig models. Masako et al. (27) showed that splenomegaly and bacterial burden in the spleen were undetectable in 1 × 10^5^ of NMII bacteria-infected Wt, B cell-deficient, NK cell-deficient, TNFα-deficient, and IFNγ-deficient mice at 28 days postinfection. Although 1 × 10^5^ of NMII bacteria can induce splenomegaly and bacterial burden in T cell-deficient mice, the splenomegaly and bacterial numbers were significantly lower than those of virulent NMI-infected mice, indicating that the avirulent NMII strain will be cleared at 28 days postinfection. Additionally, only very high doses (10^8^) of NMII can induce splenomegaly in severe combined immune deficient (SCID) mice, which lacks complete adaptive immunity (28). These data indicate that NMII bacteria possess the potential to be a safe and effective live attenuated vaccine against virulent *C. burnetii* infection (25, 29). In general, live attenuated vaccines are highly efficient as they mimic the natural infection in the host without causing pathogenicity (30). Many successful live bacterial vaccines have been approved by the FDA and are used extensively for decades to prevent various respiratory (BCG) and enteric (cholera) pathogenic diseases (31). Considering the advantages of live attenuated vaccines, non-pathogenic NMII bacteria might be useful for the development of a live attenuated vaccine against Q fever.

In this study, we examined if viable avirulent NMII bacteria can elicit protective immunity against virulent *C. burnetii* infection in mouse models of Q fever. Interestingly, viable NMII bacteria elicited a similar level of long-term protection against various virulent *C. burnetii* infections as the PIV in mice. In addition, viable NMII bacteria-induced protective immunity depends on both B cells and T cells, but T cells may play a critical role in controlling bacterial replication. Thus, this study demonstrates the feasibility of using avirulent NMII viable bacteria as a live attenuated vaccine against human Q fever.

## Material and Methods

### Bacterial Strains

*C. burnetii* Nine Mile phase I (NMI) clone 7 (RSA 493), Nine Mile phase II (NMII) clone 4 (RSA 439), Scurry phase I (Q177), and Priscilla phase I (Q217) were propagated in acidified citrate cysteine medium-D (ACCM-D), as previously described (32). Bacteria were purified by centrifugation at 15,000 × *g* for 30 min, followed by two washes with sterile 1× phosphate-buffered saline (PBS), and stored at -80°C until use. All phase I virulent strains used in this study undergo four passages and handled under biosafety level-3 conditions at the UTSA.

### Bacterial Quantification

Briefly, 200 μl of lysis buffer (1 M Tris, 0.5 M EDTA, 7 mg/ml glucose, 28 mg/ml lysozyme) and 10 μl of proteinase K (20 mg/ml) were added to 10 µl of bacterial stock and incubated at 60°C for 18 h. Next, 21 μl of 10% SDS was added to the samples and incubated at room temperature for 1 h. Finally, DNA was extracted using a High Pure PCR Template Preparation Kit (Roche, Indianapolis, IN) as directed by the manufacturer. Bacterial load was determined using TaqMan assay by quantifying *C. burnetii* com1 (FAM) (Custom Plus TaqMan™ RNA Assay, Invitrogen). The standard curve was generated using recombinant plasmid DNA (com1 gene ligated into pBluescript vector), and the results are represented genome equivalents (GE). Further, the bacterial viability was verified by serially diluting 10 µl of bacterial stock and plating in ACCM-D agarose plates and observing the colony-forming units by *C. burnetii* after 7–10 days as previously described (32).

### Formalin-Killed Bacteria and Vaccine Formulation

Purified *C. burnetii* NMI and NMII were inactivated for 48 h in 10% formalin, followed by dialysis in deionized water. Antigen concentrations were then measured using a BCA protein assay kit (Pierce, Rockford, IL) per the manufacturer’s instructions, and the concentration was represented as micrograms (µg). Ten micrograms of formalin-killed PIV and PIIV was dissolved in 50 µl PBS and mixed with 50 µl of alum adjuvant. The formulation was mixed well for 15 min.

### Animal

Eight-week-old female BALB/c mice, B cell KO (*Ighm^tm1Cgn^*) mice, T cell KO (*Foxn1^nu^*) mice, CD4 KO (*Cd4^tm1Mak^*), and CD8 KO (*Cd8a^tm1Mak^*) mice were purchased from the Jackson Laboratory (Bar Harbor, ME). Since female mice are more resistant than males to *C. burnetii* infection, female mice were exclusively used in this study. All mice were housed in sterile microisolator cages containing four mice per cage under pathogen-free conditions at the UTSA animal biosafety level 3 laboratory facility. Animals were fed normally as per University regulations. All research protocols used in this study were approved by the Institutional Biosafety Committee and the Animal Care and Use Committee of the UTSA (MU-CP001).

### Immunization Methods

Mice were immunized with viable NMII *via* intraperitoneal, intranasal, intramuscular, or subcutaneous routes. Mice were anesthetized with isoflurane (5% induction, 1% to 2% maintenance) delivered in oxygen by using an anesthetic vaporizer before each immunization and challenge experiment. Since ≥10^7^ of bacteria were used as the optimized infectious dose for all virulent NMI infections in mice (33) and to avoid unnecessary immune activation, we used 10^5^ GE of NMII as immunization dose in all immunization experiments if not specifically mentioned.

#### Intraperitoneal Immunization (IP)

10^1^, 10^3^, 10^5^, and 10^7^ GE of NMII bacteria were dissolved in 400 µl PBS and injected using a sterile 25-G needle into the lower right quadrant of the animal’s abdomen at the 30° angle to a depth of 0.5 cm.

#### Intranasal Immunization (IN)

Using a sterile micropipette, 10^5^ GE of NMII bacteria were dissolved in 20 µl PBS and slowly released into the nostrils.

#### Intramuscular Immunization (IM)

10^5^ GE of NMII bacteria was dissolved in 20 µl PBS and injected into the caudal thigh of the right pelvic leg to a depth of approximately 2 to 4 mm.

#### Subcutaneous Immunization (SQ)

Subcutaneous immunization was used to compare the protective efficacy of 10^5^ GE of viable NMII with 10 µg of formalin-killed PIV and PIIV. For subcutaneous injection, mice were restrained by scruff and the formulated vaccines were injected into the tent of skin over the scruff using a sterile 25-G needle to a depth of 0.5 cm.

### Serum and Splenocyte Isolation for Adoptive Transfer

Serum and splenocytes were collected from viable NMII-immunized mice and transferred to naive mice, respectively. Blood was collected through cardiac puncture from euthanized animal, incubated for 30 min at room temperature, and centrifuged at 1,500 × *g* for 10 min at room temperature, and the serum was collected. Splenocytes were isolated from BALB/c mice at 14 days post vaccination. Spleens were removed and homogenized. The cell suspension was then filtered through a 100-μm-pore-size nylon mesh to remove any connective tissue. Splenocytes were pelleted by centrifugation at 500 × *g* for 8 min and suspended in 5 ml of ammonium chloride–potassium (ACK) lysis buffer for 5 min at room temperature to lyse red blood cells. Remaining cells were then pelleted by centrifugation at 500 × *g* for 8 min and resuspended in 2 ml of fluorescence-activated cell sorting (FACS) buffer (PBS supplemented with 0.5% bovine serum albumin [BSA], 2 mM EDTA, and 0.1% sodium azide) for counting. Splenocytes (1 × 10^7^) suspended in 100 μl of PBS were intraperitoneally (IP) injected into each mouse, while 500 μl of pooled serum was injected into each mouse. Three days after adoptive transfer, immune serum- or splenocyte-receiving and control mice were IP challenged with 1 × 10^7^ GE of NMI and observed for 14 days.

### *C. burnetii* Challenge and Necropsy

For *C. burnetii* challenge, virulent NMI bacteria grown in ACCM-D were used. Mice were anesthetized and 10^7^ GE of NMI bacteria were dissolved in 400 µl PBS and was IP injected. Body weight was measured at 0, 3, 7, 10, and 14 days post-challenge. Mice were euthanized by CO_2_ exposure at 14 days post NMI challenge. Spleen was dissected and weighed, and 40 mg of spleen was used for genomic quantification. Blood was collected by cardiac puncture technique, and serum was separated by spinning blood at 1,500 × *g* for 10 min at room temperature. If not processed immediately, spleen and serum were stored at -20°C for further use.

### qPCR

Spleen pieces were homogenized in 200 μl of lysis buffer (1 M Tris, 0.5 M EDTA, 7 mg/ml glucose, 28 mg/ml lysozyme) and filtered through a 100-μm-pore-size nylon mesh to remove any connective tissue. Ten microliters of proteinase K (20 mg/ml) was added to each sample prior to incubation at 60°C for 18 h. Next, 21 μl of 10% SDS was added to samples and incubated at room temperature for 1 h. Finally, DNA was extracted using a High Pure PCR Template Preparation Kit (Roche, Indianapolis, IN) as directed by the manufacturer. Bacterial burden was determined using TaqMan assay by quantifying the *C. burnetii com1* or *CBU_0680* gene and normalized using the mouse *tfrc* gene. Custom Plus TaqMan™ RNA Assay, FAM for *com1*, or *CBU_0680* was designed and procured from Invitrogen. TaqMan™ Copy Number Reference Assay, mouse, *tfrc* (VIC) was used to quantify the *tfrc* genes in the mouse genome. The experiment was conducted using an Applied Biosystems QuantStudio3 real-time PCR system. The standard curve was generated using recombinant plasmid DNA (*com1*, *CBU_0680*, *mouse tfrc* genes ligated into the pBluescript vector), and the results are represented as log_10_ com1 gene copy number. All the experiments are performed at least in triplicates including both technical and biological replicates.

### *C. burnetii* NMI-Specific ELISA

Sera from vaccinated and unvaccinated control mice were used for quantification of total IgM, IgG, IgG1, IgG2a, and IgG3 subclass antibodies. Microtiter plates (96-well) were coated with 100 μl of inactivated NMI antigen (0.5 μg/ml) or unlabeled anti-IgM or -IgG antibody (0.5 μg/ml, for the standard curve) (Southern Biotech, Birmingham, AL) in 0.05 M carbonate/bicarbonate coating buffer (pH 9.6) for 24 h at 4°C. Plates were blocked with 1% BSA in PBS-T buffer (0.05% Tween 20 in 1× PBS) and then incubated for 2 h with 200 μl of diluted sample serum (1:200 to 1:1,200) or serially diluted pure IgM, IgG, IgG1, IgG2a, or IgG3 (Southern Biotech) at room temperature. Plates were washed four times with PBS-T buffer and then incubated with 100 μl of diluted horseradish peroxidase (HRP)-conjugated goat anti-mouse IgM, IgG, IgG1, or IgG2a (1:4,000 to 1:8,000) (Southern Biotech) at room temperature for 1 h. Plates were washed again four times with PBS-T, followed by the addition of 100 μl of 3,3′,5,5′-tetramethylbenzidine (TMB) substrate (Thermo Fisher Scientific). Reactions were stopped using 1 M H_3_PO_4_, and absorbance was measured at 450 nm using an Infinite F50 (Tecan, Switzerland) microplate reader.

### Statistical Analysis

Statistical analysis of body weight loss, splenomegaly, and *C. burnetii* load in spleen was determined by Welch’s unpaired t-test using GraphPad Prism 9.00 software (GraphPad). For all analyses, p < 0.05 was deemed significant.

## Results

### Viable Avirulent NMII Bacteria Interfere Virulent NMI Infection

**Figure 1:**
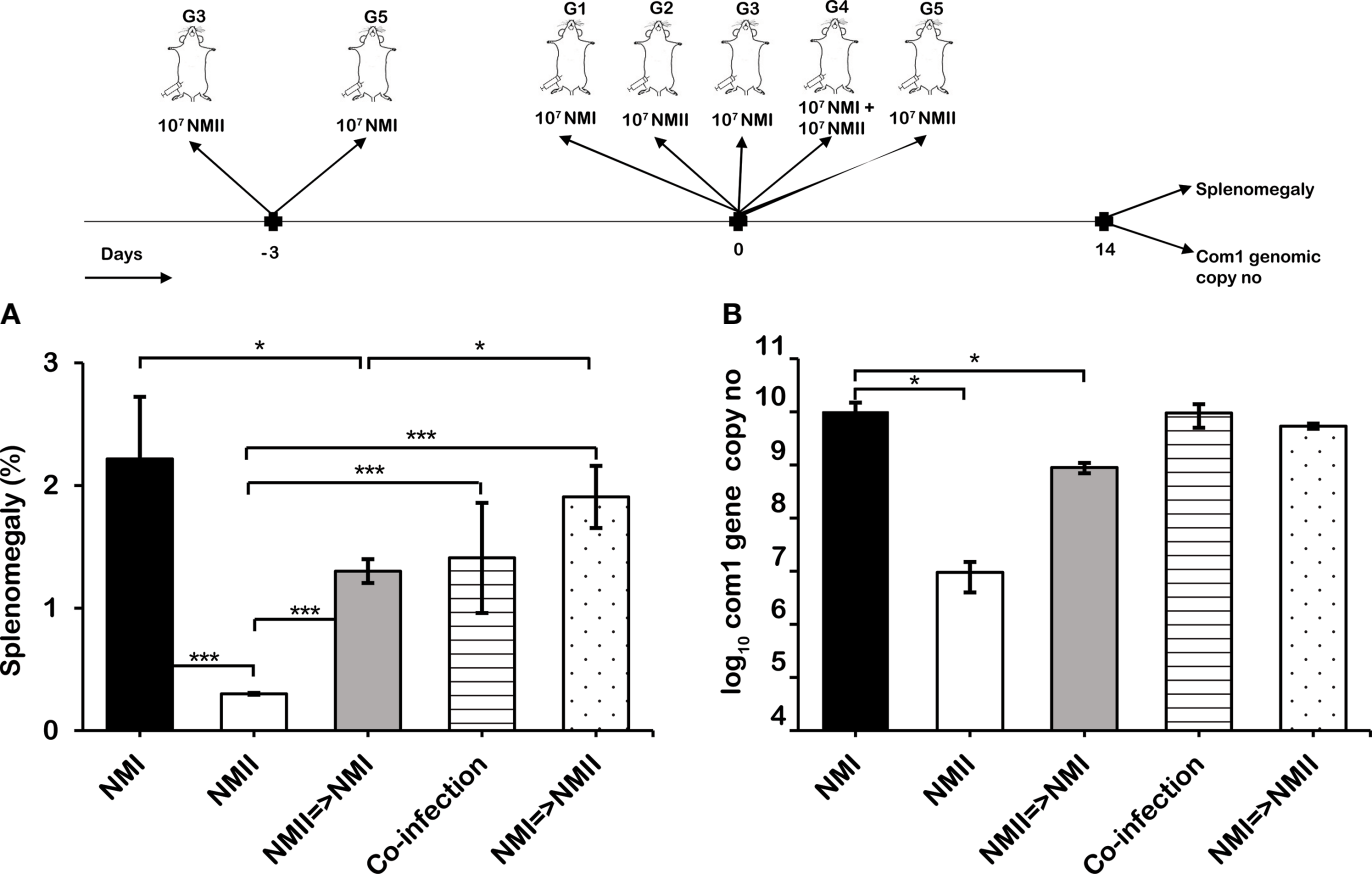
Viable avirulent NMII bacteria interfering with virulent C. burnetii NMI infection. BALB/c mice were IP infected with 1) 1 × 10^7^ virulent NMI, 2) 1 × 10^7^ avirulent NMII, 3) 1 × 10^7^ NMII 3 days before infection with 1 × 10^7^ NMI, 4) 1 × 10^7^ NMI + 1 × 10^7^ NMII, or 5) 1 × 10^7^ NMI 3 days before infection with 1 × 10^7^ NMII. Splenomegaly **(A)** and bacterial burden in the spleen **(B)** were evaluated at 14 days postinfection (dpi). Splenomegaly is expressed as percent of spleen weight/body weight. Bacterial burden was determined by real-time quantitative PCR (qPCR) and is expressed as log_10_ *C. burnetii com1* genomic copy numbers. Each experimental group includes four mice, with error bars representing the standard deviations from the means. *p < 0.05; ***p < 0.001, as determined by Welch t test using GraphPad.

To explore whether viable avirulent NMII can interfere the infection caused by the virulent NMI, we examined i) if coinfection of mice with NMI and NMII would significantly affect the ability of NMI to cause disease and ii) if priming of mice with alive NMII before infection with NMI would alter the infection caused by the virulent NMI. Eight-week-old BALB/c mice were IP infected with 1) 1 × 10^7^ virulent NMI, 2) 1 × 10^7^ avirulent NMII, 3) 1 × 10^7^ NMII 3 days before infection with 1 × 10^7^ NMI, 4) 1 × 10^7^ NM I +1 × 10^7^ NMII, or 5) 1 × 10^7^ NMI 3 days before infection with 1 × 10^7^ NMII. Splenomegaly and bacterial burden in the spleen were measured at 14 days post *C. burnetii* infection. As shown in **Figure 1A**, compared to NMI-infected mice, splenomegaly was significantly reduced in NMI and NMII-coinfected mice and mice infected with avirulent NMII 3 days before infection with virulent NMI. In addition, splenomegaly in mice infected with NMI 3 days before infection with NMII was similar to that in NMI-infected mice. However, the bacterial burden was similar in the spleens among NMI-infected mice regardless of infection with or without NMII bacteria (**Figure 1B**). These results indicated that NMII bacteria interfered with the severity of NMI infection-induced disease but did not affect NMI bacterial replication in mice. Interestingly, the findings suggest that the viable non-pathogenic NMII bacteria may be capable of inducing protective immunity against virulent *C. burnetii* infection.

### Mouse Immunization With Viable NMII Bacteria Conferred Significant Protection Against Virulent NMI Challenge

**Figure 2:**
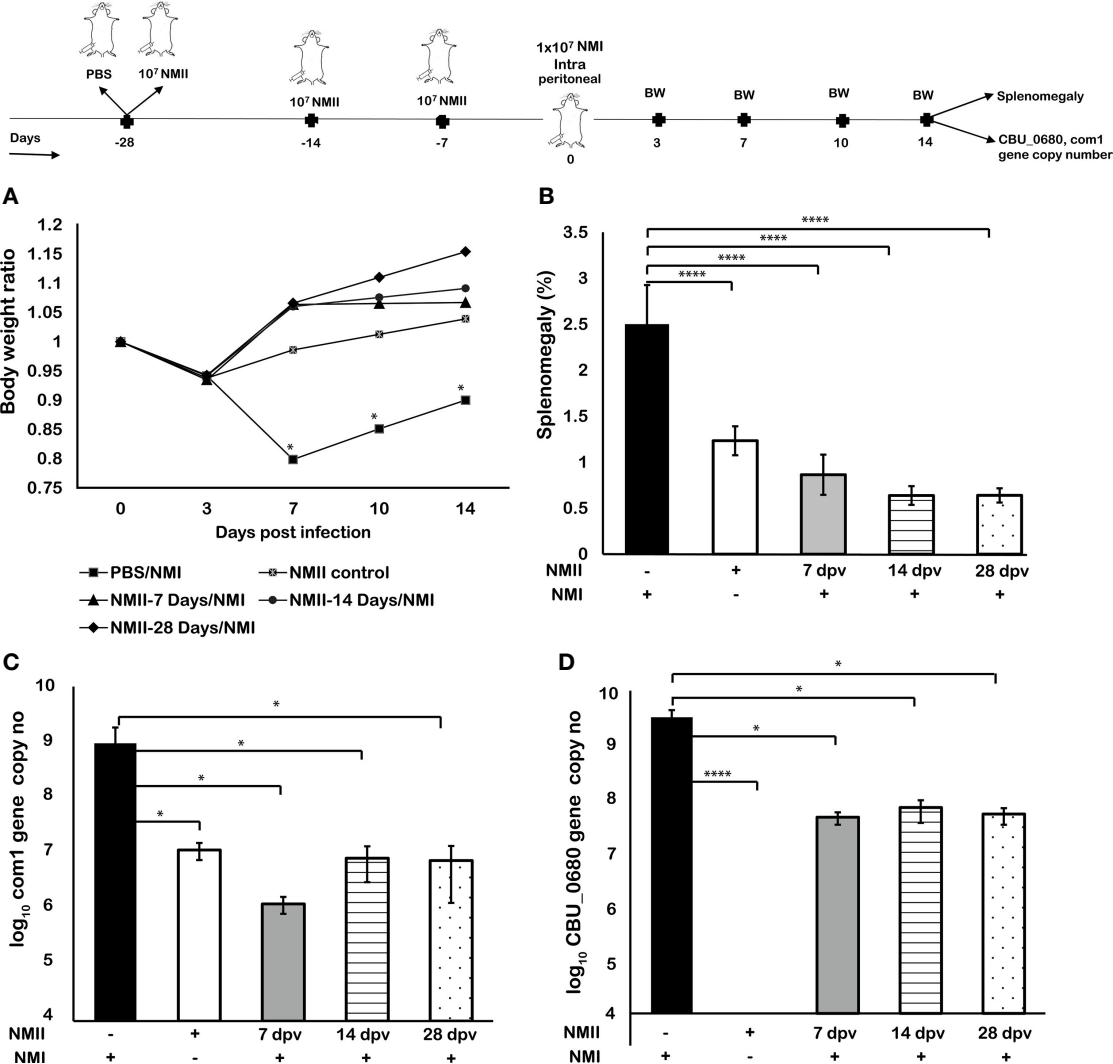
*Mouse immunization with viable NMII bacteria conferred significant protection against virulent NMI challenge.* BALB/c mice were IP vaccinated with 1 × 10^7^ GE of viable NMII and IP challenged with 1 × 10^7^ GE of NMI at 7, 14, and 28 days post vaccination (dpv), respectively. Body weight ratio **(A)**, splenomegaly [**(B)**, % of spleen weight/body weight], and bacterial burden in the spleen [**(C)**, log_10_ *C. burnetii com1* gene copy numbers, **(D)**, NMI-LPS-specific *CBU_0680* gene copy numbers] were evaluated at 14 dpi. Each experimental group includes four mice, with error bars representing the standard deviations from the means. *p < 0.05; ****p < 0.0001, as determined by Welch t test using GraphPad.

To confirm whether viable non-pathogenic NMII can induce protective immunity against virulent NMI infection, we examined if mouse immunization with viable avirulent NMII bacteria would provide significant protection against virulent NMI challenge. Three groups of BALB/c mice were IP immunized with 1 × 10^7^ viable NMII bacteria and challenged with virulent NMI bacteria at 7, 14, and 28 days post-immunization with NMII, respectively. In addition, mice were infected with 1 × 10^7^ NMI or NMII bacteria and served as controls. Splenomegaly and bacterial burden in the spleen were measured at 14 days post virulent NMI challenge. As shown in **Figure 2A**, compared to NMI-infected control mice, NMII-immunized mouse-protected NMI infection induced body weight loss at all the time points post-immunization. In addition, splenomegaly and bacterial burden (com1 gene copy number) were significantly reduced in NMII-immunized mice at different times post challenge with virulent NMI (**Figures 2B, C** **)**. Further, to determine the NMI-specific genomic copy number, the *CBU_0680* gene, which is not present in NMII, was quantified using RT-PCR. The results showed that the *CBU_0680* genomic copy number is similar to the *com1* genomic copy number (**Figure 2D**), further supporting that virulent NMI bacterial burden in the spleens is significantly reduced in all NMII-immunized mice regardless of the challenge time with virulent NMI. These results indicated that mice immunized with viable NMII bacteria were able to provide significant protection against virulent *C. burnetii* NMI challenge as early as 7 days post immunization, suggesting that viable non-pathogenic NMII bacteria may be useful as a live attenuated vaccine against human Q fever.

### Mice Immunized With Lower Doses of Viable NMII Bacteria Also Conferred Significant Protection Against Virulent *C. burnetii* Challenge

**Figure 3:**
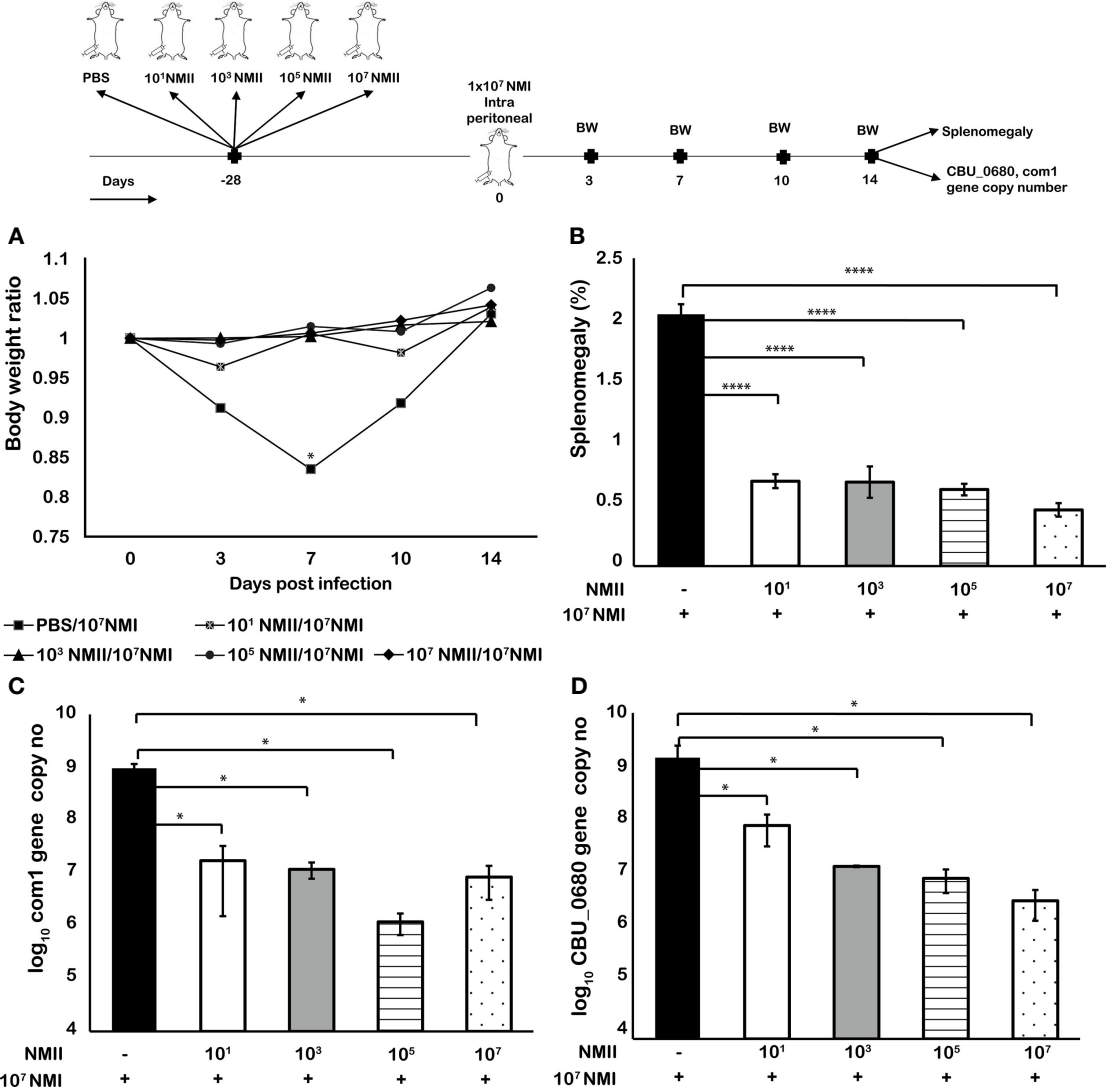
Mice immunized with lower doses of viable NMII bacteria also conferred significant protection against virulent C. burnetii challenge. BALB/c mice were IP vaccinated with 10^1^, 10^3^, 10^5^, or 10^7^ GE of NMII and IP challenged with 1 × 10^7^ GE of NMI at 28 dpv. Body weight ratio **(A)**, splenomegaly [**(B)**, % of spleen weight/body weight], and bacterial burden in the spleen [**(C)**, log_10_ *C. burnetii* com1 gene copy numbers, **(D)**, NMI-LPS-specific CBU_0680 gene copy numbers] were evaluated at 14 dpi. Each experimental group includes four mice, with error bars representing the standard deviations from the means. *p < 0.05; ****p < 0.0001, as determined by Welch t test using GraphPad.

To determine if immunization dose would affect the ability of non-pathogenic NMII bacteria to confer protection against virulent NMI infection, we tested whether mouse immunization with different doses of viable NMI bacteria would affect their ability to confer protection against virulent NMI challenge. Eight-week-old BALB/c mice were IP immunized with 1) 1 × 10^1^, 2) 1 × 10^3^, 3) 1 × 10^5^, or 4) 1 × 10^7^ of viable NMII bacteria and IP challenged with 1 × 10^7^ virulent NMI bacteria at 28 days post immunization with avirulent NMII. In addition, mice were infected with 1 × 10^7^ NMI and served as unimmunized controls. Splenomegaly and bacterial burden in the spleen were measured at 14 days post virulent NMI challenge. As shown in **Figure 3A**, compared to unimmunized control mice, all doses of NMII-immunized mice protected NMI infection-induced transitional body weight loss at 3, 7, and 10 days post NMI infection. In addition, splenomegaly and bacterial burden were significantly reduced in NMII-immunized mice regardless of the dose of immunization (**Figures 3B–D**). The lowest dose of 1 × 10^1^ viable NMI bacteria-immunized mice also provided significant protection against virulent NMI challenge. These results further demonstrated that mice immunized with viable NMII bacteria could confer significant protection against virulent *C. burnetii* challenge.

### Viable NMII Bacteria-Induced NMI-Specific Antibody Response in Mice

**Figure 4:**
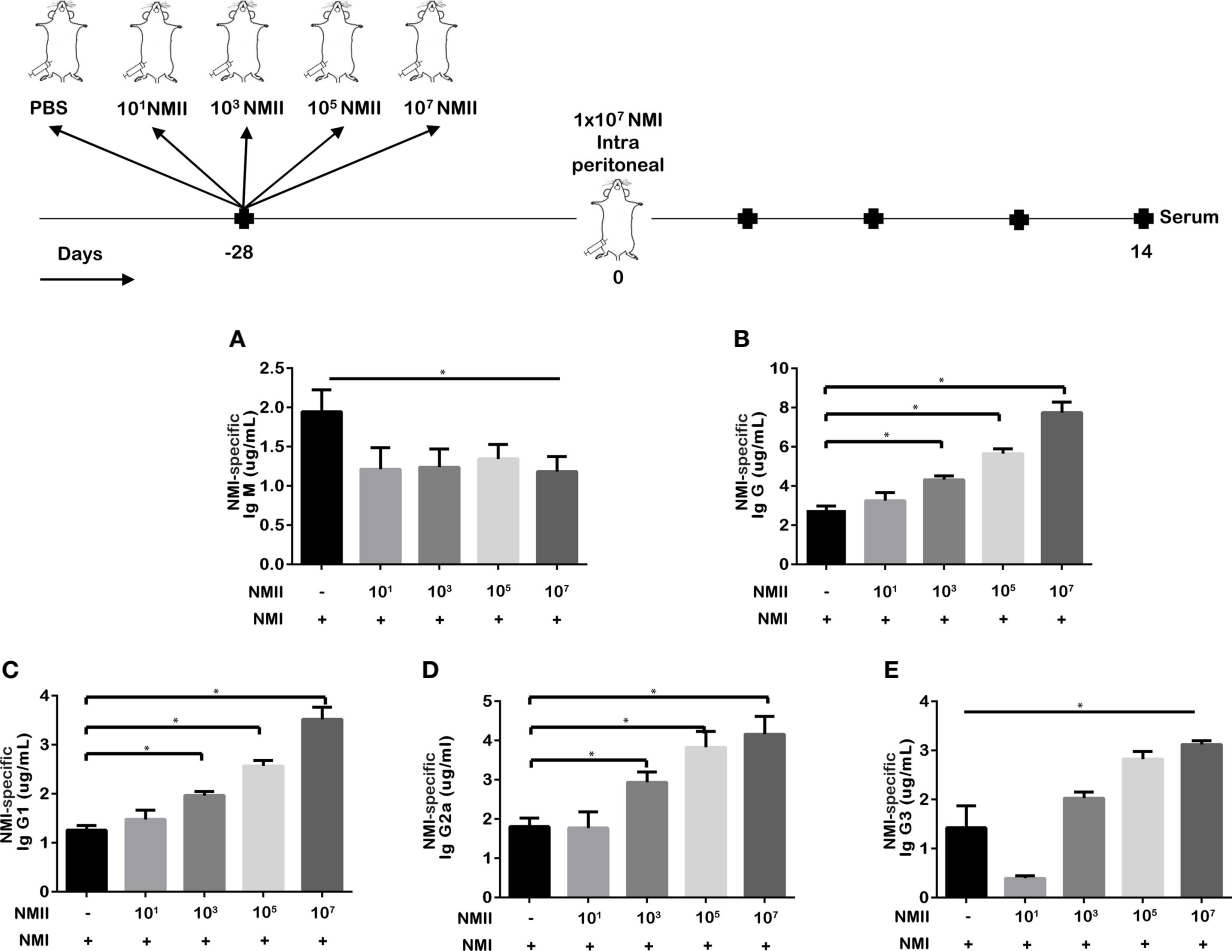
*Viable NMII bacteria induce antibody response in mice against NMI antigens.* The concentrations of *C. burnetii* NMI-specific IgM **(A)**, IgG **(B)**, IgG1 **(C)**, IgG2a **(D)**, and IgG3 **(E)** in serum samples from different doses of viable NMII-vaccinated mice and challenged with NMI at 14 dpi were analyzed by ELISA. Antibody concentration is expressed as µg/mL. Statistical analysis was performed between the NMII-immunized and unimmunized groups. *p < 0.05, as determined by Welch t test using GraphPad.

To determine if viable NMII bacteria would induce NMI-specific antibody response, immune sera were collected from mice immunized with 1) 1 × 10^1^, 2) 1 × 10^3^, 3) 1 × 10^5^, or 4) 1 × 10^7^ of viable NMII bacteria at 14 days post-challenge with NMI in the above experiment for measuring the concentrations of anti-NMI-specific IgM and IgG by ELISA. As shown in **Figure 4A**, NMI-specific IgM titer in mice immunized with live NMII bacteria was significantly lower than that in unimmunized mice, but there was no significant difference among different doses of NMII-immunized mice. In contrast, as shown in **Figure 4B**, NMI-specific IgG titer was significantly higher in mice immunized with NMII except the lowest dose (10^1^) of NMII and the pattern was similar for subclasses IgG1 and IgG2a (**Figures 4C, D** **)**. Interestingly, mice immunized with the lowest dose (10^1^) of NMII exhibited lower levels of IgG3 than all other groups including unimmunized group; however, there was no difference in protection, suggesting that IgG3 may not play a major role in NMII-mediated protection (**Figure 4E**). Collectively, these results indicated that viable NMII bacteria induced a significant NMI-specific antibody response. Notably, although the lowest dose of 1 × 10^1^ NMII-immunized mice induced a lower level of NMI-specific IgG response, they conferred a similar level of protection against NMI infection as other higher doses of NMII-immunized mice (**Figures 3B, C** **)**, suggesting that NMI-specific IgG response may not correlate with viable NMII bacteria-induced protection.

### Mouse Immunization With Viable NMII *via* Different Routes Conferred Similar Protection Against Virulent NMI Challenge

**Figure 5:**
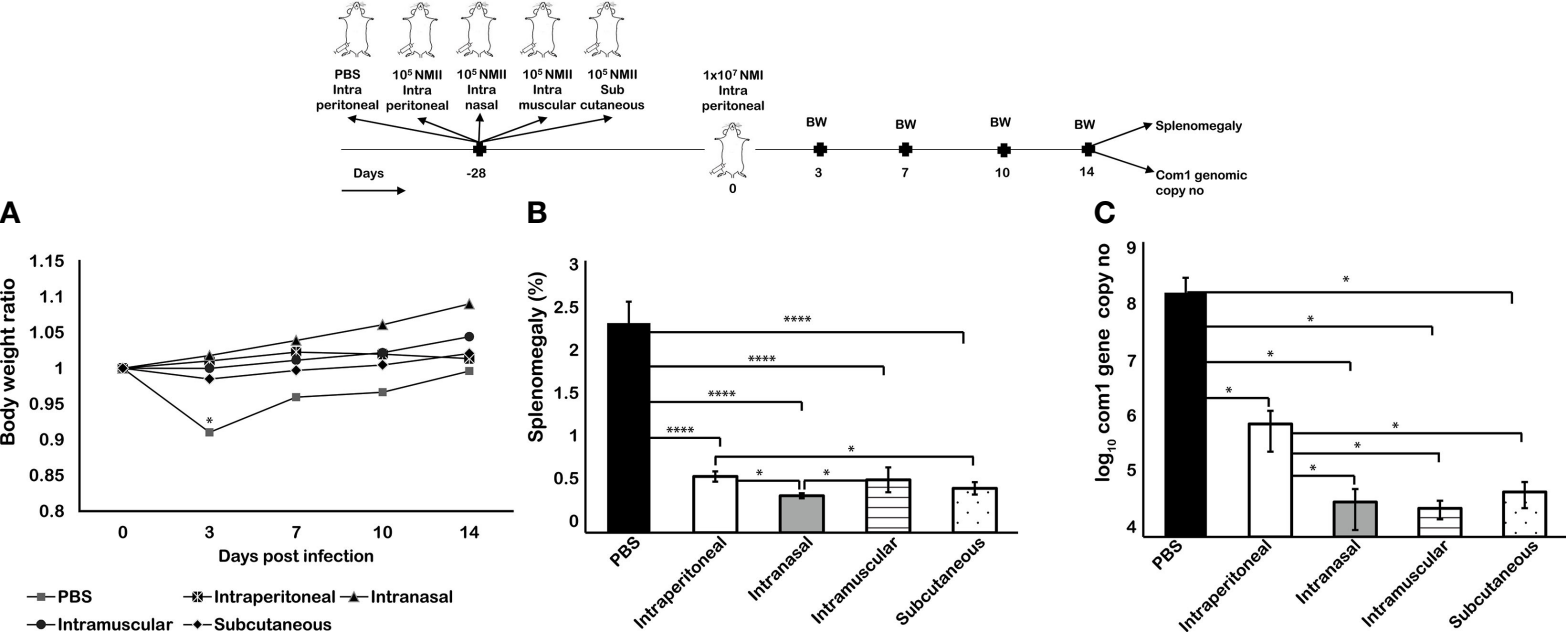
Mouse immunization with viable NMII via different routes conferred similar protection against virulent NMI challenge. BALB/c mice were vaccinated with 10^5^ GE of viable NMII *via* IP, IN, IM, or SQ routes and IP challenged with 1 × 10^7^ GE of NMI at 28 dpv. Body weight ratio **(A)**, splenomegaly [**(B)**, % of spleen weight/body weight], and bacterial burden (log_10_ *C. burnetii com1* gene copy numbers) in the spleen **(C)** were evaluated at 14 dpi. Each experimental group includes four mice, with error bars representing the standard deviations from the means. *p < 0.05; ****p < 0.0001, as determined by Welch t test using GraphPad.

To identify the optimized route of immunization for viable NMII bacteria vaccine, we examined if the route of immunization would affect the ability of viable NMII bacteria to confer protection against virulent NMI infection. BALB/c mice were IP, IN, IM, or SQ immunized with 1 × 10^5^ of viable NMII bacteria. PBS-vaccinated mice were maintained as unimmunized control. Twenty-eight days post vaccination, mice were IP challenged with 1 × 10^7^ of virulent NMI bacteria. Splenomegaly and bacterial burden in the spleen were measured at 14 days post NMI challenge. As shown in **Figure 5A**, compared to unimmunized controls, all immunized mice protected the NMI infection-induced transitional body weight loss. Splenomegaly and bacterial burden were also significantly reduced in NMII-immunized mice regardless of the route of immunization (**Figures 5B, C** **)**. In addition, compared to IP-immunized mice, splenomegaly and bacterial burden were significantly lower in IN- and SQ-immunized mice. Although there was no significant difference in splenomegaly between IP- and IM-immunized mice, bacterial burden in IM-immunized mice was significantly lower than IP-immunized mice. Collectively, the results indicated that IN immunization with viable NMII induced a stronger protective immunity against NMI infection than the other immunization routes. Since the IN immunization route is less invasive than IP, IM, and SQ immunization, it would be the optimized immunization route for human vaccination with live NMII vaccine.

### Viable NMII Bacteria Conferred a Similar Level of Protection as the Formalin-Killed NMI Vaccine (PIV)

**Figure 6:**
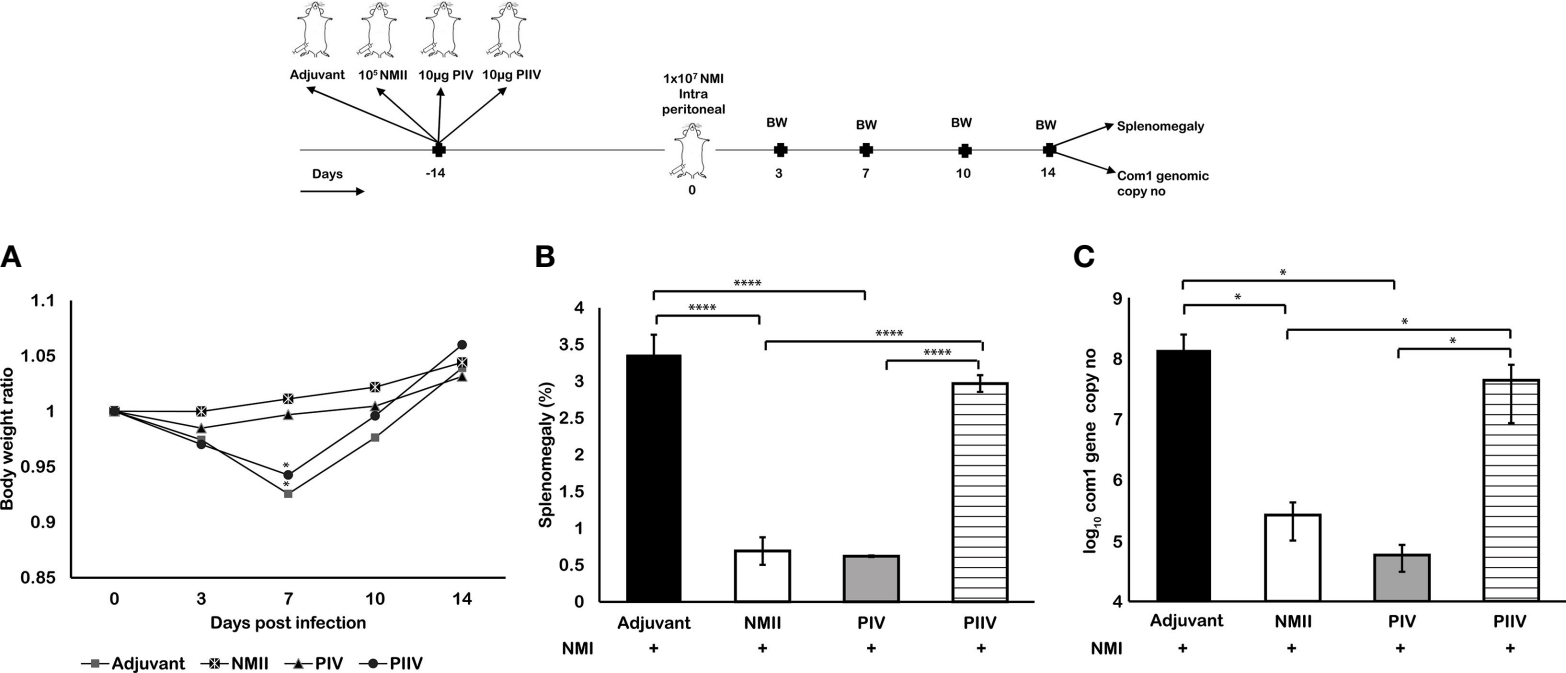
Mouse immunization with viable NMII bacteria provides similar protection as formalin-killed PIV. BALB/c mice were IP vaccinated with 1 × 10^5^ GE of viable NMII, SQ immunized with 10 µg of PIV, or SQ immunized with 10 µg of PIIV and IP challenged with 1 × 10^7^ GE of NMI at 14 dpv. Body weight ratio **(A)**, splenomegaly (**B**, % of spleen weight/body weight), and bacterial burden (log_10_ *C. burnetii com1* gene copy numbers) in the spleen **(C)** were evaluated at 14 dpi. Each experimental group includes four mice, with error bars representing the standard deviations from the means. *p < 0.05; ****p < 0.0001, as determined by Welch t test using GraphPad.

To determine if avirulent NMII live vaccine can confer a similar level of protection against virulent *C. burnetii* infection as the current licensed vaccine, Q-Vax, we compared the protective efficacy between viable NMII bacteria and PIV in BALB/c mice *via* SQ immunization. As shown in **Figure 6A**, compared to adjuvant and formalin-killed NMII vaccine-immunized mice, both viable NMII-immunized and PIV-immunized mice were able to protect NMI infection-induced transitional body weight loss at 7 days post challenge and significantly reduced the splenomegaly (**Figure 6B**) and bacterial burden in the spleens (**Figure 6C**) at 14 days post challenge at a comparable level. These results indicated that viable NMII bacteria induced a similar level of protection against virulent NMI infection as the PIV. In addition, the observation that viable NMII bacteria conferred significant protection against virulent NMI infection but killed NMII vaccine did not confer a measurable protection, suggesting that bacterial replication in mice may be required for viable NMII-induced protective immunity.

### Viable NMII Bacteria Conferred a Long-Term Protection Against Virulent NMI Infection

**Figure 7:**
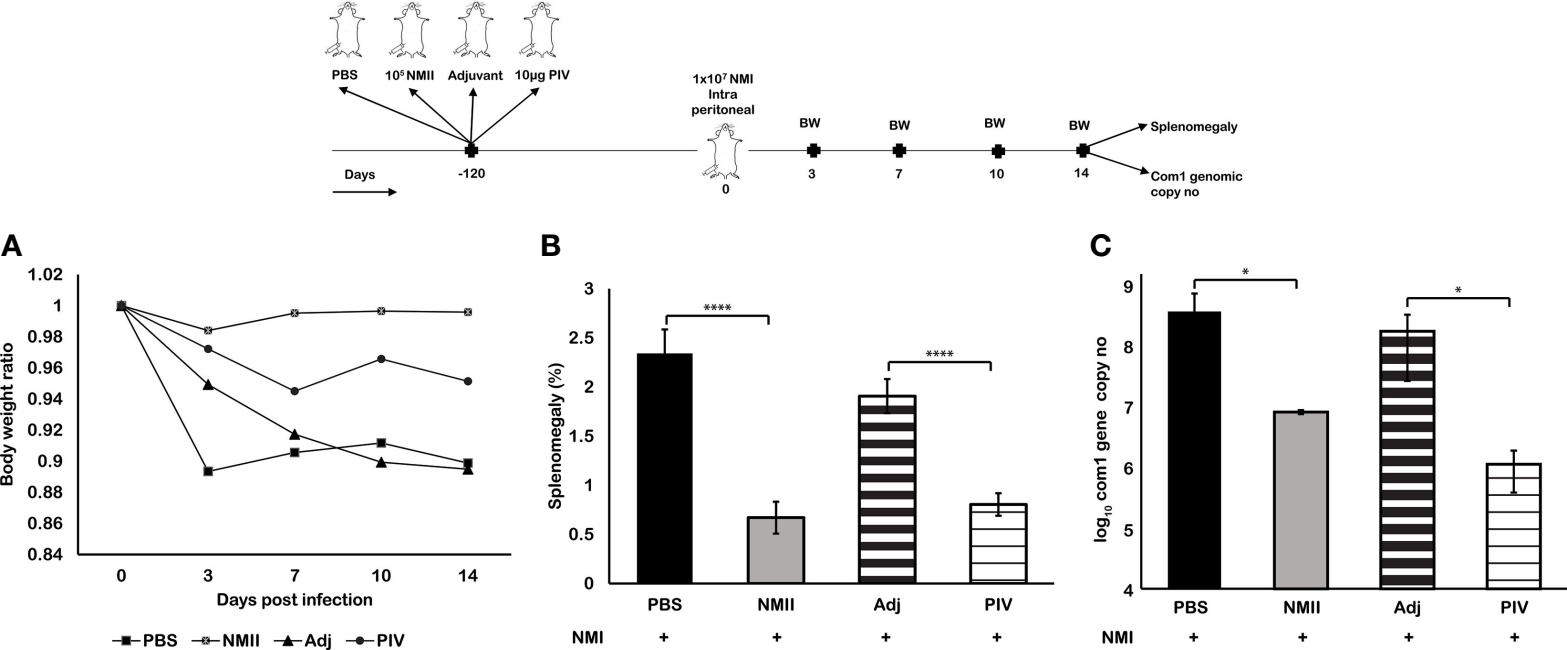
Mouse immunization with viable NMII bacteria conferred long-term protection against virulent NMI challenge. BALB/c mice were SQ vaccinated with 10^5^ GE of viable NMII; 10 µg of PIV, PBS, or adjuvant; and IP challenged with 10^7^ GE NMI at 120 dpv. Body weight ratio **(A)**, splenomegaly (**B**, % of spleen weight/body weight), and bacterial burden (log_10_ *C. burnetii com1* gene copy numbers) in the spleen **(C)** were evaluated at 14 dpi. Each experimental group includes four mice, with error bars representing the standard deviations from the means. *p < 0.05; ****p < 0.0001, as determined by Welch t test using GraphPad.

According to our previous studies, 10 µg formalin-killed PIV provided the highest levels of protection. To determine whether viable NMII bacteria can confer a similar level of long-term protection as the PIV, we compared the protective efficacy against virulent NMI infection between 10^5^ GE of viable NMII bacteria and 10 µg of PIV in BALB/c mice at 120 days post SQ immunization. As shown in **Figure 7**, compared to unvaccinated and adjuvant-immunized mice, both viable NMII-immunized and PIV-immunized mice were able to protect NMI infection-induced significant body weight loss at different times post challenge (**Figure 7A**) and significantly reduced the splenomegaly (**Figure 7B**) and bacterial burden in the spleens (**Figure 7C**) at 14 days post challenge at a comparable level. In addition, there was no significant difference in splenomegaly and bacterial burden in the spleens between viable NMII-immunized and PIV-immunized mice. These results demonstrated that viable NMII bacteria induced a similar level of long-term protection against virulent NMI challenge as the PIV. Notably, a nodule-like inflammatory response was observed at the site of injection in PIV-immunized mice but the abnormal response at the site of injection did not appear in viable NMII-immunized mice. This observation indicates that immunization with viable NMII bacteria unlikely will induce adverse reactions as the PIV, suggesting that viable NMII bacteria might be useful as a live attenuated vaccine to replace the existing Q-Vax for prevention of human Q fever.

### Viable NMII Bacteria Conferred a Significant Cross-Protection Against Various Virulent *C. burnetii* Isolates

**Figure 8:**
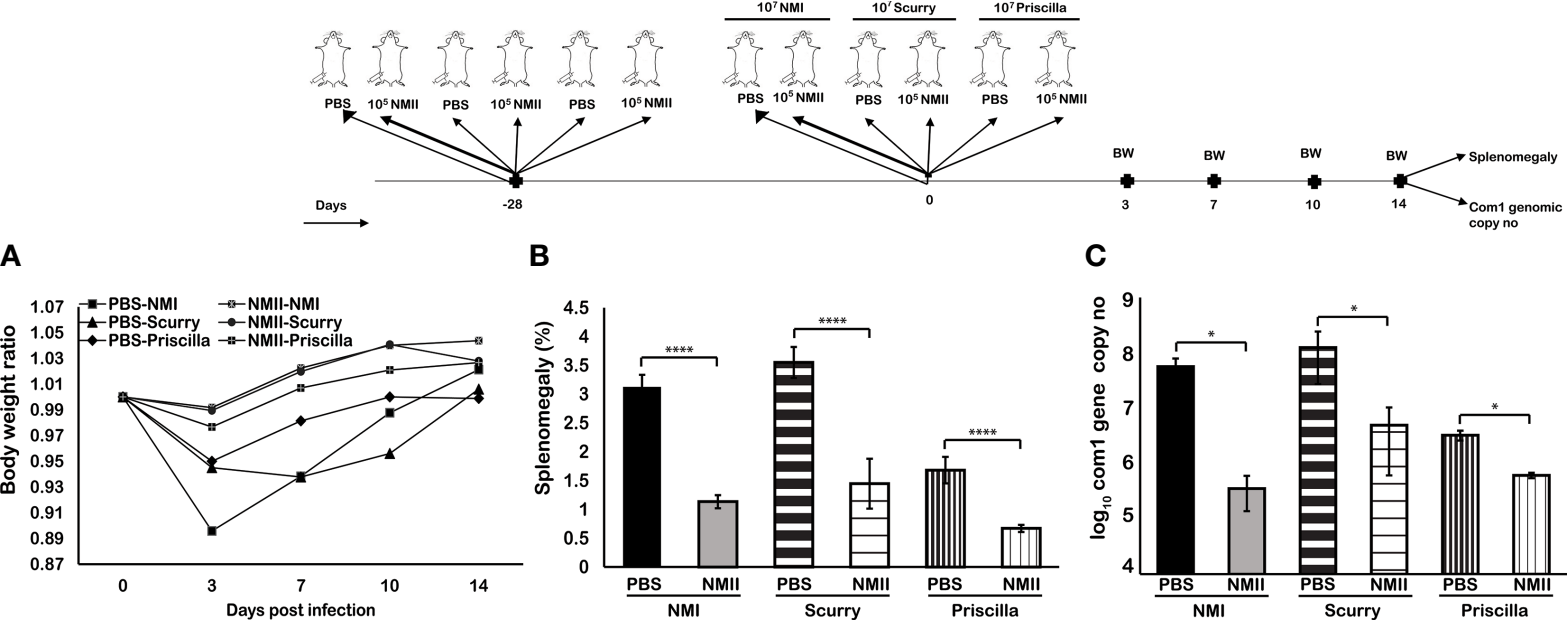
Mice immunized with viable NMII bacteria confer protection against other virulent strains of *C. burnetii.* BALB/c mice were IP vaccinated with 1 × 10^5^ GE of viable NMII and IP challenged with 1 × 10^7^ GE of NMI, Scurry, or Priscilla at 28 days dpv. Body weight ratio **(A)**, splenomegaly [**(B)**, % of spleen weight/body weight], and bacterial burden (log_10_ *C. burnetii com1* gene copy numbers) in the spleen **(C)** were evaluated at 14 dpi. Each experimental group includes four mice, with error bars representing the standard deviations from the means. *p < 0.05; ****p < 0.0001, as determined by Welch t test using GraphPad.

The results from the above experiments demonstrate that viable NMII bacteria can elicit long-lasting significant protection against challenge with virulent NMI strains. However, it remains unknown if viable NMII bacteria can confer cross-protection against challenge with other virulent *C. burnetii* isolates. To address this question, we evaluated the cross-protective efficacy of viable NMII bacteria against virulent *C. burnetii* Priscilla and Scurry strains, which are chronic Q fever-associated isolates. Three groups of BALB/c mice were IN immunized with 1 × 10^5^ of viable NMII bacteria and IP challenge with 1 × 10^7^ of virulent NMI, Priscilla, or Scurry bacteria at 28 days post-immunization. In addition, three groups of PBS-immunized mice were infected with 1 × 10^7^ of NMI, Priscilla, or Scurry bacteria and used as infection control for each respective strain. Splenomegaly and bacterial burden in the spleen were measured at 14 days after virulent *C. burnetii* infection. As shown in **Figure 8**, compared to unimmunized and *C. burnetii*-infected control mice, viable NMII-immunized mice were able to protect NMI, Priscilla, and Scurry infection-induced transitional body weight loss at 3 days post challenge (**Figure 8A**) and significantly reduced the splenomegaly (**Figure 8B**) and bacterial burden in the spleens (**Figure 8C**) at 14 days post challenge. These results demonstrated that IN immunization of mice with viable NMII bacteria conferred significant protection against various virulent *C. burnetii* isolate infection, suggesting that viable NMII bacteria can confer broad protection against challenge with heterogeneous isolates of virulent *C. burnetii*.

### Immune Sera and Splenocytes From Viable NMII-Immunized Mice Provided a Significant Protection Against NMI Challenge in Naive Mice

**Figure 9:**
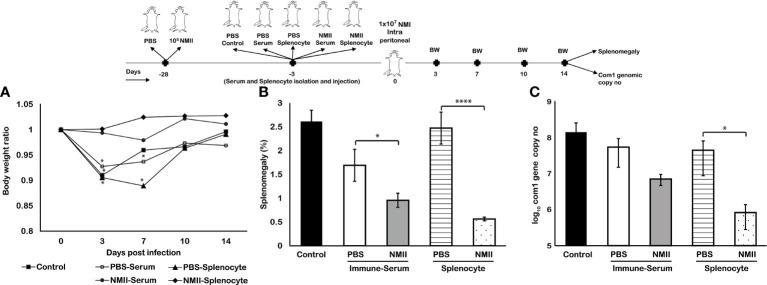
Adoptive transfer of serum and splenocytes from NMII-immunized mice induces protection against NMI challenge in naïve mice. BALB/c mice were IP transferred with sera or splenocytes from PBS or viable NMII-immunized mice and IP challenged with 1 × 10^7^ GE of NMI at 3 days post adoptive transfer. Body weight ratio **(A)**, splenomegaly [**(B)**, % of spleen weight/body weight], and bacterial burden (log_10_ *C. burnetii com1* gene copy numbers) in the spleen **(C)** were evaluated at 14 dpi. Each experimental group includes four mice, with error bars representing the standard deviations from the means. *p < 0.05; ****p < 0.0001, as determined by Welch t test using GraphPad.

To determine the roles of humoral and cellular immunity in viable NMII-induced protection, we tested if adoptive transfer of immune sera or splenocytes from NMII-vaccinated mice can confer protection in naive mice against NMI infection. Immune sera and splenocytes were isolated from PBS or 1 × 10^5^ of viable NMII bacteria-immunized BALB/c mice at 14 days post immunization and transferred to naive BALB/c mice by IP injection, respectively. Immune sera, splenocytes, or PBS receiving mice were challenged with 1 × 10^7^ of NMI bacteria at 3 days after adoptive transfer. Splenomegaly and bacterial burden in the spleen were measured at 14 days post NMI challenge. Compared to mice that received PBS, sera, or splenocytes from PBS-immunized mice, mice receiving immune sera or splenocytes from NMII-immunized mice were able to protect NMI infection-induced significant body weight loss at 3 and 7 days post challenge (**Figure 9A**) and significantly reduced the splenomegaly (**Figure 9B**). In addition, compared to mice that received PBS, sera, or splenocytes from PBS-immunized mice, the bacterial burden in the spleens was significantly reduced in mice receiving splenocytes from NMII-immunized mice but was similar in mice receiving immune sera from NMII-immunized mice (**Figure 9C**). These results suggest that both humoral and cellular immunities are involved in the viable NMII-induced protection but cellular immunity may play a critical role in controlling bacterial replication in mice.

### Viable NMII Bacteria-Induced Protective Immunity Depends on Both B Cells and T Cells, but T Cells May Play a Critical Role in Controlling Bacterial Replication

**Figure 10:**
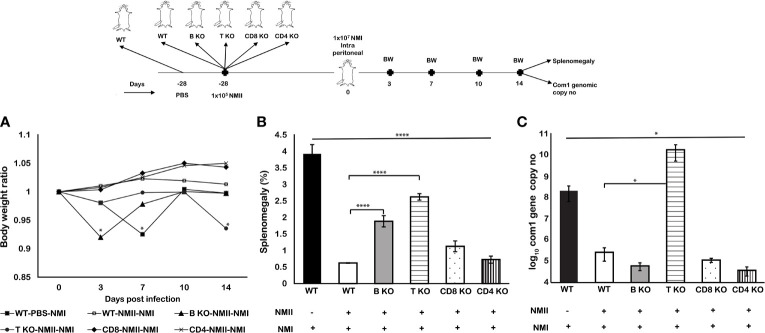
Viable NMII bacteria induced partial protection in B cell and T cell knockout mice against virulent NMI challenge. C57BL/6J wild-type (WT) and B cell-, T cell-, CD4^+^ T cell-, or CD8^+^ T cell-deficient mice were IP immunized with 10^5^ GE of viable NMII and IP-challenged with 1 × 10^7^ GE of NMI at 28 dpv. Additionally, PBS-immunized WT mice were IP challenged with 1 × 10^7^ GE of NMI served as unimmunized controls. Body weight ratio **(A)**, splenomegaly [**(B)**, % of spleen weight/body weight], and bacterial burden (log_10_ *C. burnetii com1* gene copy numbers) in the spleen **(C)** were evaluated at 14 dpi. Each experimental group includes four mice, with error bars representing the standard deviations from the means. *p < 0.05; ****p < 0.0001, as determined by Welch t test using GraphPad.

To further understand the mechanisms of viable NMII bacteria-induced protective immunity, we examined if B cell, T cell, CD4^+^ T cell, or CD8^+^ T cell deficiency in mice will significantly affect the ability of viable NMII bacteria to confer protection against virulent NMI infection. C57BL/6J wild-type (WT) and B cell-, T cell-, CD4^+^ T cell-, or CD8^+^ T cell-deficient mice were IP immunized with viable NMII bacteria and challenged with NMI at 28 days post-immunization. Additionally, naive WT mice were infected with NMI and served as unimmunized controls. Splenomegaly and bacterial burden in the spleen were examined at 14 days post challenge. As shown in **Figure 10A**, NMI infection induced a significant body weight loss in unimmunized WT and NMII-immunized B cell-deficient and T cell-deficient mice at different times post challenge, but there was no body weight loss in NMII-immunized WT, CD4^+^ T cell-deficient, and CD8^+^ T cell-deficient mice. In addition, compared to unimmunized WT mice, NMI infection-induced splenomegaly was significantly reduced in all NMII-immunized mice (**Figure 10B**). However, although splenomegaly was similar between NMII-immunized B cell-deficient and T cell-deficient mice, it was significantly higher than NMII-immunized WT, CD4^+^ T cell-deficient, and CD8^+^ T cell-deficient mice. As shown in **Figure 10C**, the bacterial burden was comparable in the spleens from NMII-immunized WT, B cell-deficient, CD4^+^ T cell-deficient, and CD8^+^ T cell-deficient mice, but it was significantly higher in the spleen from NMII-immunized T cell-deficient mice. Notably, the bacterial burden in the spleen from NMII-immunized T cell-deficient mice was even significantly higher than that in NMI-infected unimmunized WT mice. Collectively, these results demonstrated that viable NMII bacteria conferred a comparable level of protection against virulent *C. burnetii* challenge in WT, CD4^+^ T cell-deficient, and CD8^+^ T cell-deficient mice, and a partial protection in B cell-deficient and T cell-deficient mice. The observation that the bacterial burden in the spleens from NMII-immunized T cell-deficient mice was significantly higher than that in NMI-infected unimmunized WT mice suggests that T cells may play a critical role in controlling virulent *C. burnetii* replication in mice. Thus, viable NMII bacteria-induced protective immunity depends on both B cells and T cells, but T cells may play a critical role in controlling bacterial replication.

## Discussion

Although formalin-killed *C. burnetii* phase I whole-cell vaccine is highly effective for the prevention of Q fever in humans and animals (8–10), its wide usage is limited by the adverse reactions at the site of inoculation, especially in subjects with preexisting immunity to *C. burnetii*. To overcome this problem, it is necessary to develop alternative vaccines that can avoid the adverse effects during vaccination. Recently, Scholzen et al. (34) identified HLA class II epitopes, which show long-lived immunoreactivity in naturally infected individuals, making them desirable candidates for a novel human multi-epitope Q fever vaccine. Xiong et al. (35) showed that attenuated *Listeria monocytogenes* vaccine vectors are an efficient antigen-delivery platform that can be used to induce robust protective CD8^+^ T cell-immune responses against *C. burnetii* infection. Gilkes et al. (36) formulated *C. burnetii* antigens with a novel TLR triagonist adjuvant platform, offering an alternative platform for *C. burnetii* vaccine development. Our previous study demonstrated the protective efficacy of lipopolysaccharide-targeted peptide mimic vaccine against Q fever (12). However, these alternative vaccine candidates were unable to confer the same level of protection against virulent *C. burnetii* infection as the PIV. It remains a significant challenge for developing a safe and effective alternative vaccine that can confer a similar level of protection against Q fever as the PIV but without inducing the adverse reactions.

Utilizing a live attenuated vaccine that expresses the similar antigenic determinants as the virulent *C. burnetii* but lacks the virulence factors, which reduces the risk of infection while providing maximum protection, might be an ideal paradigm for safe vaccination against human Q fever. *C. burnetii* NMII strains derived from NMI exhibit truncated LPS, fulfilling the criteria required for a live attenuated vaccine candidate. Because of the genomic deletion of LPS biosynthetic genes, NMII bacteria produce truncated LPS, lacking the major virulence factor (15). NMII infection in a guinea pig model did not induce splenomegaly or fever response, indicating that NMII is a non-pathogenic strain (25). Our previous study also demonstrated that the infection rate of neutrophils by NMII is similar to that of NMI, yet the intracellular bacterial load of NMII is significantly less than that of NMI, showing that NMII is more readily cleared from neutrophils than NMI (37). Additionally, proteomic analysis has indicated that NMII and NMI respond differentially to stress in L929 cells, indicating that the intracellular survival of both strains is distinct (38). Regarding protective immunity, NMII infection induces toll-like receptor 4-independent dendritic cell maturation with high IL-12 and TNF production (29), implying that NMII might activate dendritic cell-mediated T-cell response in the host. These findings suggest that viable NMII bacteria may be able to activate the immune system against subsequent infections with virulent *C. burnetii* isolates without causing clinical illness. Thus, this study aimed to investigate the feasibility of using viable NMII bacteria as a live attenuated vaccine against virulent *C. burnetii* infection in mice.

It is notable that mice immunized with viable NMII bacteria at 3 days prior to NMI challenge significantly reduced splenomegaly but were unable to protect against bacterial replication. These results may be due to *C. burnetii* being an intracellular pathogen, which can establish a vacuolar biogenesis after invading host cells for its intracellular replicating, resulting in bacteria escaping from the host immune system at the early stage of infection (39, 40). However, mice immunized with viable NMII bacteria provided significant protection against NMI infection-induced splenomegaly and bacterial replication from 7 days up to 120 days post vaccination with the trend showing that protection increases over time. These results suggest that viable NMII bacteria were able to confer significant protection against NMI challenge and protection can last at least 4 months tested. It is important to note that immunization using different doses of NMII indicated a similar level of protection regardless of the immunization dose and that even a very low infection dose of 10^1^ GE was sufficient to induce protection. Collectively, these findings demonstrate the possibility of using avirulent NMII stain as a live attenuated vaccine against human Q fever.

The major problem with the existing vaccine, Q-VAX^®^, is that subcutaneous or intra-dermal injection of the vaccine induces a strong inflammatory response at the site of inoculation, especially in individuals who have been exposed to *C. burnetii* prior to vaccination (9). An adverse events following immunization (AEFI) study conducted in Q-VAX^®^ vaccinated veterinary, and animal science students, at Australian universities showed that Q-VAX^®^ induced severe local and systemic AEFI (41). These data provide strong evidence to support that Q-VAX^®^ has the potential to induce AEFI in humans. Similar to those observations from human vaccinees, we have also observed nodule-like inflammation reactions at the sites of PIV-injected mice; however, the adverse reactions were not observed in the site of SQ-immunized mice with viable NMII bacteria (data not shown). These observations suggest that a viable NMII bacteria-based vaccine is potentially safer than PIV for immunization of humans. However, further studies needed to examine whether viable NMII bacteria can induce local and systemic adverse reactions in *C. burnetii* sensitized animals.

Generally, mucosal vaccines can induce both systemic and mucosal immunity, including antigen-specific IgA response, especially at mucosal surfaces, which are the frontlines of host defense against multiple pathogens (42). Additionally, the intranasal vaccination method is a less invasive route for immunization of humans and has been a widely approved vaccination route for human vaccines. Interestingly, we found that IN immunization with viable NMII induced a stronger protective immunity against NMI infection than IP, IM, and SQ immunization with NMII. Our previous study demonstrated that even high doses of NMII infection (aerosol infection with 1 × 10^9^ of bacteria) did not induce significant inflammatory response and cause clinical disease in immunodeficient SCID mice (37). These data suggest that IN immunization is potentially safe and effective for human vaccination with the NMII live attenuated vaccine. To further validate the efficacy of viable NMII vaccine by IN immunization, we examined if mice IN vaccinated with viable NMII bacteria would provide similar levels of broad protection as the PIV against virulent *C. burnetii* Priscilla and Scurry strain challenge. The results indicate that IN immunization with viable NMII bacteria conferred a similar level of significant protection as the PIV against challenge with both virulent Priscilla and Scurry strains. Since both Priscilla and Scurry strains are considered as chronic Q fever-associated isolates, the observation that viable NMII bacteria conferred significant protection against both Priscilla and Scurry strains provided further evidence to support that viable NMII bacteria can confer broad protection against both acute and chronic Q fever-associated isolates. Additionally, this result further demonstrates that IN immunization would be a safe, effective, and practicable vaccination method for using viable NMII bacteria as an attenuated live vaccine against human Q fever.

Vaccination is an active immune approach for simulation of the immune system to generate antigen-specific humoral and cellular immune responses, which can induce lasting-protective immunity against infectious diseases (43). The major advantage of using a whole-cell vaccine is that it induces a long-term T cell response, in addition to antibody response (44). Izzo et al. (45) reported that Q-VAX^®^ induces a long-lived T-cell response which is detectable for at least 8–10 years following vaccination. To understand the mechanism of the NMII live attenuated vaccine-induced protective immunity, we examined if transfer immune sera or splenocytes from viable NMII bacteria-vaccinated mice would protect naive recipient mice against virulent NMI infection. The results indicated that both immune sera and splenocytes from NMII-immunized mice provided a similar level of protection against NMI infection-induced body weight loss and splenomegaly. However, although splenocytes from NMII-immunized mice conferred significant protection against NMI infection-induced bacterial burden in the spleens, immune sera from NMII-immunized mice were unable to provide protection against the bacterial burden in naive recipient mice. These results suggest that both humoral and cellular immunities are involved in the viable NMII-induced protection, but cellular immunity may play a critical role in controlling of bacterial replication in mice.

To identify which host immune components are crucial for viable NMII bacteria-induced protective immunity, we examined if B cell, T cell, CD4^+^ T cell, or CD8^+^ T cell deficiency in mice will significantly affect the ability of viable NMII bacteria to confer protection against virulent NMI infection. The results demonstrated that viable NMII bacteria conferred a comparable level of protection against virulent NMI challenge in WT, CD4^+^ T cell-deficient, and CD8^+^ T cell-deficient mice, and a partial protection in B cell-deficient and T cell-deficient mice. In addition, the result that the bacterial burden in the spleens from NMII-immunized T cell-deficient mice was significantly higher than that in NMI-infected unimmunized WT mice suggests that T cells may play a critical role in controlling virulent *C. burnetii* replication in mice. Collectively, these results suggest that viable NMII bacteria-induced protective immunity depends on both B cells and T cells but T cells may play a critical role in controlling bacterial replication. Interestingly, our previous study (33) also demonstrated that PIV conferred i) comparable levels of protection against NMI infection in WT, CD4^+^ T cell-deficient, and CD8^+^ T cell-deficient mice; ii) partial protection against splenomegaly but no protection against bacterial replication in T cell-deficient mice; and iii) no measurable protection in B cell-deficient mice. These data suggest that the mechanisms of viable NMII vaccine and PIV-induced protection may be similar and highlight the role of T cells for controlling bacterial replication in vaccine-induced protective immunity against this intracellular bacterial pathogen. In addition, our recent study (31) demonstrated that MHC-II restricted PIV-specific CD4^+^ T cells and Th1 immune response plays a crucial role in PIV-mediated protection against *C. burnetii* infection. These studies (14, 31, 44) suggest that anti-PI specific Abs plays an important role in protection from the development of clinical disease at an early stage against *C. burnetii* infection, while the T cell-mediated Th1 immune response is critical for clearance and complete elimination of the organisms at the late stage of the infection. Therefore, novel vaccine approaches for Q fever should be focused on boosting both humoral and cellular immune responses.

In summary, our results demonstrated that viable NMII bacteria elicited a similar level of long-term protection against various virulent *C. burnetii* infections as the PIV in mice. In addition, viable NMII bacteria-induced protective immunity depends on both B cells and T cells but T cells may play a critical role in controlling bacterial replication. Thus, this study provided the first evidence to demonstrate the feasibility of using avirulent NMII viable bacteria as a live attenuated vaccine against human Q fever.

## Data Availability Statement

The raw data supporting the conclusions of this article will be made available by the authors, without undue reservation.

## Ethics Statement

The animal study was reviewed and approved by the Institutional Biosafety Committee and the Animal Care and Use Committee of the UTSA.

## Author Contributions

Conceived and designed the experiments: VK, SA, YZ, and GZ. Performed the experiments: VK and SA. Analyzed the data: VK, SA, YZ, and GZ. Wrote the paper: VK and GZ. All authors contributed to the article and approved the submitted version.

## Funding

NIH/NIAID grant R01AI134681, R21AI130347, and R21AI137504 to GZ.

## Conflict of Interest

The authors declare that the research was conducted in the absence of any commercial or financial relationships that could be construed as a potential conflict of interest.

## Publisher’s Note

All claims expressed in this article are solely those of the authors and do not necessarily represent those of their affiliated organizations, or those of the publisher, the editors and the reviewers. Any product that may be evaluated in this article, or claim that may be made by its manufacturer, is not guaranteed or endorsed by the publisher.

## Abbreviations

NMI, Nine Mile Phase I; NMII, Nine Mile Phase II; PIV, formalin-inactivated NMI; PIIV, formalin-inactivated NMII; KO, knockout; LPS, lipopolysaccharides.
